# Defective Vagal Innervation in Murine *Tbx1* Mutant Hearts

**DOI:** 10.3390/jcdd5040049

**Published:** 2018-09-23

**Authors:** Amélie Calmont, Naomi Anderson, Jenifer P. Suntharalingham, Richard Ang, Andrew Tinker, Peter J. Scambler

**Affiliations:** 1 INSERM UMRS 1155, Centre for Kidney Research, 4 Rue de la Chine, 75020 Paris, France; 2 UCL Great Ormond Street-Institute of Child Health, 30 Guilford Street, London WC1N 1EH, UK; n.anderson@ucl.ac.uk (N.A.); j.suntharalingham@ucl.ac.uk (J.P.S.); p.scambler@ucl.ac.uk (P.J.S.); 3 William Harvey Heart Centre, Barts & The London School of Medicine & Dentistry, Queen Mary University of London, London EC1M 6BQ, UK; r.ang@ucl.ac.uk (R.A.); a.tinker@ucl.ac.uk (A.T.); 4 Department of Medicine, Rayne Institute, University College London, London WC1E 6JJ, UK

**Keywords:** *Tbx1*, DiGeorge Syndrome, neural crest cells, cranial nerves, innervation

## Abstract

Haploinsufficiency of the T-box transcription factor *TBX1* is responsible for many features of 22q11.2 deletion syndrome. *Tbx1* is expressed dynamically in the pharyngeal apparatus during mouse development and *Tbx1* homozygous mutants display numerous severe defects including abnormal cranial ganglion formation and neural crest cell defects. These abnormalities prompted us to investigate whether parasympathetic (vagal) innervation of the heart was affected in *Tbx1* mutant embryos. In this report, we used an allelic series of *Tbx1* mouse mutants, embryo tissue explants and cardiac electrophysiology to characterise, in detail, the function of *Tbx1* in vagal innervation of the heart. We found that total nerve branch length was significantly reduced in *Tbx1^+/−^* and *Tbx1^neo2/−^* mutant hearts expressing 50% and 15% levels of *Tbx1*. We also found that neural crest cells migrated normally to the heart of *Tbx1^+/−^*, but not in *Tbx1^neo2^* mutant embryos. In addition, we showed that cranial ganglia IX^th^ and X^th^ were fused in *Tbx1^neo2/−^* but neuronal differentiation appeared intact. Finally, we used telemetry to monitor heart response to carbachol, a cholinergic receptor agonist, and found that heart rate recovered more quickly in *Tbx1^+/−^* animals versus controls. We speculate that this condition of decreased parasympathetic drive could result in a pro-arrhythmic substrate in some 22q11.2DS patients.

## 1. Introduction

22q11.2 deletion syndrome (22q11.2DS) is the most common microdeletion syndrome in humans associated with a typical 3MB deletion within chromosome 22 [1,2]. 22q11.2DS patients exhibit craniofacial and ear anomalies, glandular defects, dysphagia and psychiatric illnesses [3]. Congenital heart defects are the greatest cause of mortality and may comprise great vessel disruption and septation defects and/or mal-alignment of the outflow tract. *TBX1* is located within the 3Mb deleted region and haploinsufficient *Tbx1* mutations in mouse phenocopy this disorder [4,5,6]. *TBX1* is the only gene from the deleted region which has been shown to be heterozygously mutated in 22q11.2DS-like patients without a deletion and is therefore the best candidate gene for the major structural defects in 22q11.2DS patients [7]. In addition to controlling the development of pharyngeal arch, glandular and cardiac derivatives typically affected in 22q11.2DS phenotypes, we and others have shown that shown that *Tbx1* is involved in the regulation of cranial nerves and associated neural crest cell (NCC) migratory pathways [8,9]. Haploinsufficiency of *Tbx1* leads to cranial nerve fusion [10] as well as hypoplasia of the vagus nerve adjacent to the oesophagus [11].

The vagus nerve is the tenth of twelve paired cranial nerves. It conveys mostly afferent fibres with sensory functions but has also efferent components. Sensory neurones differentiate from thickened pseudostratified ectodermal placodes, and newly specified neuroblasts will delaminate from these placodes to migrate and coalesce to form ganglia. The development of epibranchial placodes, and more generally all cranial sensory ganglia, is highly dependent on how NCCs develop and migrate [12]. The nodose placode contributes to the vagus sensory ganglion, which consists of both NCC- and epibranchial placode-derived neurons [13]. Conversely, NCCs, which migrate to the dorsal aorta, differentiate into neurons and extend axonal projections into the cardiac tissue, and provide all of the efferent parasympathetic innervation to the heart [14]. In the present study, we analyse the implication of this vagal nerve deficit for the innervation of a single defined peripheral compartment: the mouse heart.

## 2. Materials and Methods

### 2.1. Mouse Strains

Mice were paired in the evening, and the morning on which a vaginal plug was observed was defined as E0.5. We used *Tbx1*^+/−^ (MGI:2179136) [5], *Tbx1^CRE^* (MGI:3757964) [15], *Rosa^YFP^* (MGI:2449038) [16] and *Tbx1*^neo2/+^ (MGI:3664784) [17] mutant alleles to generate the appropriate genotypes. All lines were created in AB2.2 ES cells (129S5/SvEvBrd background) and backcrossed into the C57Bl/6N genetic background for at least 10 generations. Animal work was conducted under Home Office Licence PPL70/7892 (UCL Great Ormond Street-Institute of Child Health, London, UK) and PPL70/7665 (Queen Mary University and University College London, London, UK). 

### 2.2. In Situ Hybridization and Immunolabeling

Samples were fixed in 4% formaldehyde in PBS and processed as 15 μm cryosections. In situ hybridisation (ISH) was performed with a digoxigenin-labeled riboprobe from a mouse *Sema3c* cDNA plasmid (gift of Jonathan Raper, University of Pennsylvania, Philadelphia, Pennsylvania, USA) as previously described [8]. Sectioned and wholemount immunolabeling was carried out as previously described [11,18] using the following primary antibodies: rabbit anti-GFP (MBL), chick anti-GFP (Aves), rabbit anti-neurofilament-66 (Millipore, Burlington, MA, USA), mouse anti-HuC/D (Invitrogen, Waltham, MA, USA), rabbit anti-Phox2b [19] and mouse anti-AP2α (Developmental Studies Hybridoma Bank, University of Iowa, Iowa, IA, USA). Secondary antibodies used were Alexa Fluor-conjugated (Thermo Fisher Scientist, Waltham, MA, USA). Neurofilament-HuC/D double stained embryos were dehydrated and cleared as previously documented [11]. Neurofilament-stained hearts were photographed using a Zeiss SteREO Lumar.V12 microscope equipped with an Axioxam HRc camera and UV lamp under a rhodopsin red filter. Neurons were traced using the software ImageJ (NIH, USA) and branch length extracted.

### 2.3. Electrocardiogram Recordings and Pharmacological Studies

Mice were studied between 10–12 weeks of age weighing between 27–47 g. Mice were anaesthetised with 1% isoflurane entrained with oxygen-enriched air delivered via a nose cone. Body temperature was maintained using a heating mat. Needle electrodes were placed in a standard lead II configuration to record surface electrocardiogram (ECG). ECG signals were sampled at 2 kHz using a multi-channel Powerlab recorder (AD Instruments, Dunedin, New Zealand) and analysed off-line using the ECG extension module of CHART 4.0 (AD Instruments). Heart rate (HR) was measured at baseline and at 30 s intervals up to 5 minutes after administration of the muscarinic agonist Carbachol (CBC, 0.1 mg kg^−1^ i.p.).

### 2.4. Isolation and Culture of NCCs

Cardiac neural crest cells were isolated and cultured as previously described [20]. Briefly, post-otic neural tube was isolated and digested in a mix of collagenase/dispase for 15 min at 37 °C. Fine dissection of neural tube tissue from somites 1–4 was achieved with sharpen tungsten needles. Dissected tissue was cultured on Fibronectin-coated microwell chamber slides (Thermo Fisher Scientist) in DMEM supplemented with 10% SVF. Explants were left for 10 days to allow full differentiation of cNCCs into parasympathetic neurons expressing the acetylcholine transporter marker (AChT, Millipore).

### 2.5. Statistical Analysis

Total nerve branch lengths are represented as mean ± SD. Statistical analysis was performed using a one-way ANOVA followed by Dunnett’s multiple comparison test between *Tbx1*^+/+^ and *Tbx1*^+/−^ on one side and *Tbx1*^+/+^ and *Tbx1^neo2^*^/−^ on the other side. For HR measurements, data are reported as individual values, means ± SEM, or as box and whiskers plots. Statistical significance of repeated measures at different time intervals was determined using mixed level modelling and statistical significance between 2 groups was determined by Mann-Whitney test. Differences between the groups with *p* < 0.05 were considered to be significant. Statistical analysis was performed using prism 5, GraphPad (San Diego, CA, USA) and IBM SPSS Statistics (New York, NY, USA) Subscription.

## 3. Results

### 3.1. Defective Cardiac Innervation in Tbx1 Mutants

**Figure 1:**
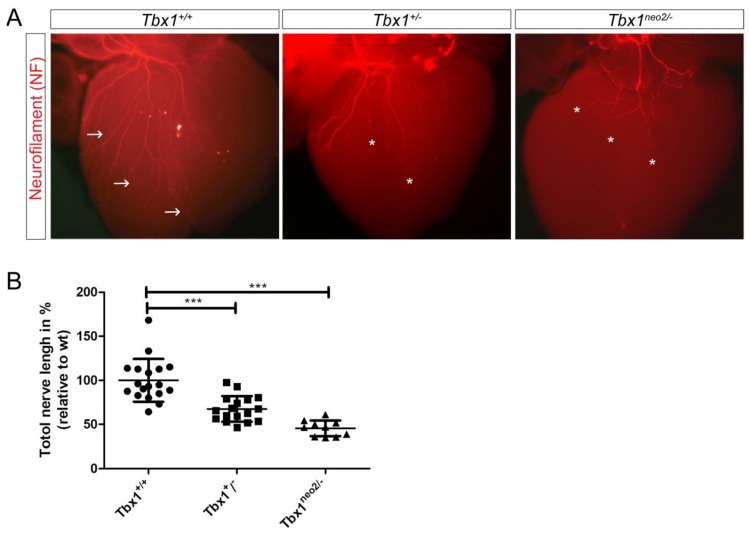
*Tbx1*^+/−^ and *Tbx1^neo2^*^/−^ have defective innervation. (**A**) Wholemount immunostaining at E15.0 for neurofilament-66 allowed visualisation of the parasympathetic innervation descending from the cardiac plexus and branching into the ventricles (white arrows). Truncation of axons was seen in *Tbx1*^+/−^ and *Tbx1^neo2^*^/−^ hearts (stars). (**B**) Quantification of axon branches on the dorsal surface of the heart in *Tbx1*^+/+^ *Tbx1*^+/−^ and *Tbx1^neo2^*^/−^, *** *p* < 0.0001, F = 31.17 for three groups, df = 2.

Intronic insertion of a selection cassette served to generate an allelic series of *Tbx1* mutant mouse models [21], which we used, in combination with the null or wildtype alleles, to generate *Tbx1^+/+^*, *Tbx1^+/−^* and *Tbx1^neo2/−^* mutant animals. *Tbx1^+/+^*, *Tbx1^+/−^* and *Tbx1^neo2/−^* embryos express 100%, 50% and 15% of the wildtype mRNA level respectively [21], allowing us to manipulate *Tbx1* expression to better model 22q11.2DS. Indeed, reducing *Tbx1* mRNA dosage to 20% makes a far better model than either the heterozygous or homozygous null mutants to study cardiac defects for instance [21]. To determine whether cardiac vagal innervation was defective in *Tbx1* mutant mice, we isolated whole hearts from *Tbx1^+/+^*, *Tbx1^+/−^* and *Tbx1^neo2/−^* mouse embryos at E15.0 and stained neuronal axon projections with anti-Neurofilament 66 (internexin neuronal intermediate filament protein, alpha) (Figure 1). At E15.0, cardiac innervation is mostly parasympathetic and reflects vagal nerve projection to the heart. Indeed, parasympathetic NCCs precede the arrival of sympathetic innervation in both avian and mammals, and is also functional before that of the sympathetic system [22,23,24]. Cardiac innervation was quantified on Neurofilament-66 wholemount immunostained embryos of the indicated genotype (Figure 1).

We observed fewer axonal branch points on the dorsal surface of the heart of E15 *Tbx1* mutant embryos compared to wildtype littermates (Figure 1A, white asterisks). The total nerve branch length covering the dorsal ventricular surface area was measured and found to be significantly reduced from a control score of 100% in *Tbx1^+/+^* (6807 μm ± 1601 μm, n=18) to 61% in *Tbx1^+/−^* (4150.3 μm ± 894.1 μm, *n* = 17) and to 49% (3399.1 μm ± 667.1 μm, *n* = 9) in *Tbx1^neo2/−^* mutants (Figure 1B).

Our data suggests that cardiac innervation is abnormal in the developing hearts of *Tbx1* mutant embryos. 

### 3.2. TBX1 Cell and Non-Cell Autonomous Role in Cardiac Innervation Defects

The lack of vagal nerve fibres innervating the heart of *Tbx1* mutants could have multiple causes and TBX1 could act at different levels, cell autonomously or non-cell autonomously, in patterning structures affected in this phenotype.

**Figure 2:**
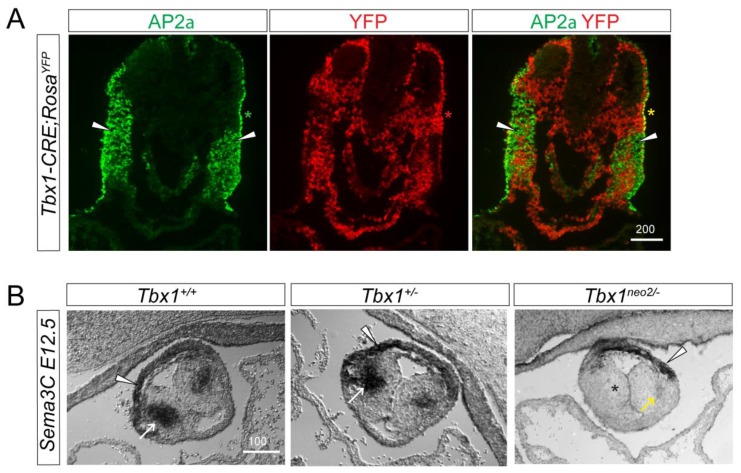
Cardiac neural crest cell defects in *Tbx1* mutant embryos. (**A**) E9.5 *Tbx1CRE-Rosa^YFP^* embryos were stained for the cNCC marker AP2α together with YFP as a marker for the *Tbx1*-lineage. cNCCs and *Tbx1* do not colocalise (arrowheads). (**B**) E12.5 *Tbx1*^+/+^, *Tbx1*^+/−^ and *Tbx1*^neo2/−^ outflow tracts were stained for the cNCC marker *Sema3C*. cNCCs have migrated into the outflow tract cushions in *Tbx1^+/+^* and *Tbx1^+/−^* but not in *Tbx1^neo2/−^* embryos. White arrowheads and arrows indicate *Sema3C* expression in myocardial cuff cells and the septal bridge area, respectively. Asterisk indicates absent *Sema3C* in the outflow tract right cushion. Yellow arrow marks residual *Sema3C*-positive cNCCs in the outflow tract left cushion. Scale Bars are in μm.

*Tbx1* is expressed in the three germ layers participating in the development of the structures affected in 22q11.2DS, but has been reported as not expressed in NCCs [25]. Cardiac NCCs (cNCCs) represent a subdivision of the cranial crest which delaminate from the neural folds in a region spanning from the otic placode to the caudal border of somite 3. They have been found to be essential for normal heart development and their ablation in the chick phenocopied most 22q11.2DS phenotypes [26]. Here, we independently examined whether any *Tbx1*-linage cells could be of NCCs origin. We used the *Tbx1*-*CRE* mouse line [15] combined with the *R26R^YFP^* allele to generate *Tbx1CRE-Rosa^YFP^* embryos. We undertook immunohistochemical analysis of *E9*.*5 Tbx1CRE-Rosa^YFP^* embryos examining the cNCC marker AP2α together with YFP as a marker for the *Tbx1*-linage. No colocalisation was found between YFP1-positive and AP2α-positive cells in the cNCC region, pointing toward a non-cell autonomous action of TBX1 on NCC patterning/migration. Of note, co-localisation of these two markers was observed in the pharyngeal surface ectoderm (PSE; Figure 2A, asterisk), as previously described [8].

cNCCs enter through the arterial pole of the heart into the developing outflow tract at E10.5. By E11.5, cNCC-derived nerves can be seen growing into the inflow of the heart, associated with the cardiac plexus, the main conduit for the parasympathetic innervation of the heart [27]. They then differentiate and extend axonal projections into the cardiac tissue. We examined the cNCC migration route within the outflow tract at E12.5 using the cNCC marker *Sema3C* [18]. Almost no *Sema3c* expression in OFT cushions was detected in *Tbx1^neo2/−^* embryos (asterisk and yellow arrow in Figure 2B), while its expression was retained in the OFT myocardium (Arrowhead, Figure 2B). 

We concluded that Tbx1, by controlling cNCC migration in a non-cell autonomous manner, is required for proper vagal innervation of the heart. An NCC migration defect and cardiac NCC deficiency observed in *Tbx1* mutant embryos could account for the loss of vagal innervation of the heart at later stages.

**Figure 3:**
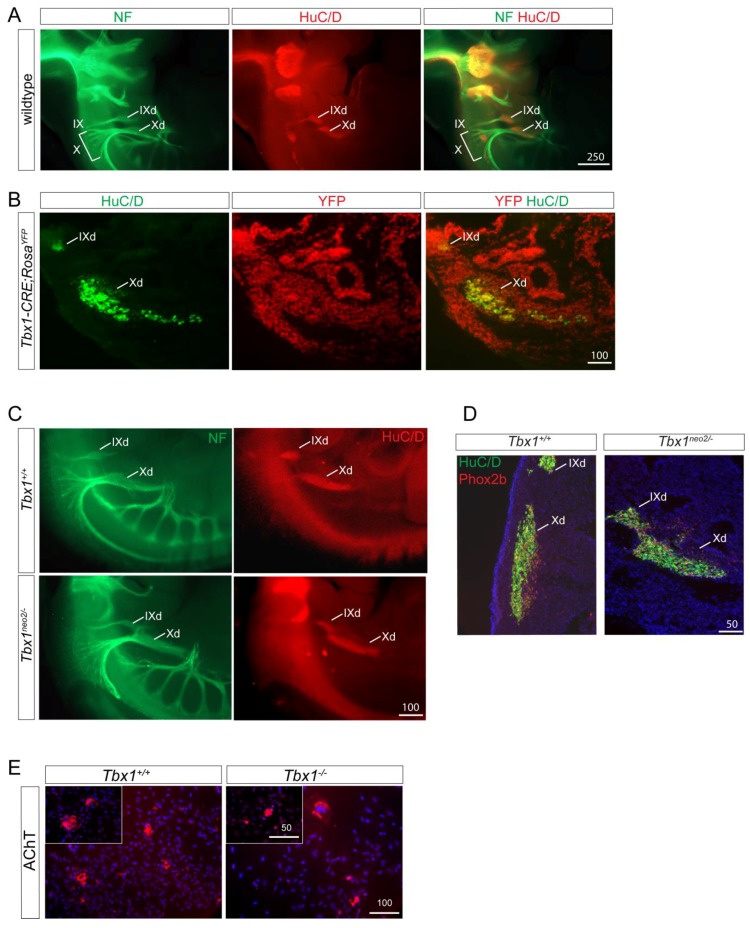
Defects in cranial ganglia in *Tbx1* mutants. (**A**) In wildtype E11.5 embryos, neurofilament labelling demonstrate aggregates of nerve forming the proximal IX^th^ and the X^th^ ganglia (IX and X in A, and nerve fibres projecting ventrally to reach distal sensory ganglia of these nerves (HuC/D positive IXd and Xd in A). (**B**) E11.5 *Tbx1CRE-Rosa^YFP^* embryos were stained for the cNCC marker AP2α together with YFP as a marker for the *Tbx1*-lineage. Co-localisation observed at the level of the IX^th^ and X^th^ ganglia. (**C**) In *Tbx1*^neo2/−^ E11.5 embryos, HuC/D neurons are in spindle shaped ganglia but are misplaced and fused. (**D**) E11.5 *Tbx1*^neo2/−^ embryos are stained positively for the neuronal marker Phox2B. (**E**) cNCCs derived from explanted neural tubes of *Tbx1*^+/−^ and *Tbx1*^−/−^ E8.75 embryos were cultured for 10 days and stained with the parasympathetic marker acetylcholine transporter (AChT). Scale bars are in μm.

*Tbx1* is expressed in the PSE and in epibranchial placodes, but not in the nodose ganglion at later stages (E10.5). In order to examine whether *Tbx1*-expressing cells can contribute to nodose ganglion neurons, we co-stained E11.5 *Tbx1CRE-Rosa^YFP^* embryos with the neuronal cell marker HuC/D and a YFP antibody. We observed overlapping marker expression in both the glossopharyngeal (IX) and nodose (X) ganglia, showing that neurons present in the nodose ganglion had previously expressed *Tbx1* (Figure 3B). Therefore, *Tbx1* could potentially play a cell autonomous role in neurogenesis, as described previously [28]. 

We next performed morphometric analyses of the IX^th^ and X^th^ ganglia using double immunohistological labelling with neurofilament-66 and HuC/D antibodies (Figure 3A,C). The IX^th^ and X^th^ ganglia of *Tbx1^+/+^* and *Tbx1^neo2/−^* were similar in appearance with HuC/D neurons located in a characteristic spindle-shaped ganglion (Figure 3C). Nevertheless, we observed a fusion between these two ganglia in *Tbx1^neo2/−^* embryos, a result which supports a placodal-positioning defect rather than a cell specific role of *Tbx1* in neuronal differentiation or survival. In agreement with this hypothesis, we found a normal distribution of the neuronal marker PHOX2b in *Tbx1^neo2/−^* embryos compared to wild type embryos (Figure 3D).

We next explanted E8.75 neural tubes from the cardiac NCC region and allowed NCC to migrate out of the tube. We cultured cNCCs for an additional 10 days and found that *Tbx1^−/−^* -derived cNCCs retain their ability to differentiate into functional parasympathetic neurons expressing the acetylcholine transporter marker AChT (Figure 3E).

Our results show that *Tbx1* controls cardiac innervation by modulating NCC and nerve migratory pathways in a non-cell autonomous fashion with no observable role in neuronal differentiation.

### 3.3. Functional Relevance of Cardiac Innervation Deficiency in Tbx1 Mutant Animals

**Figure 4:**
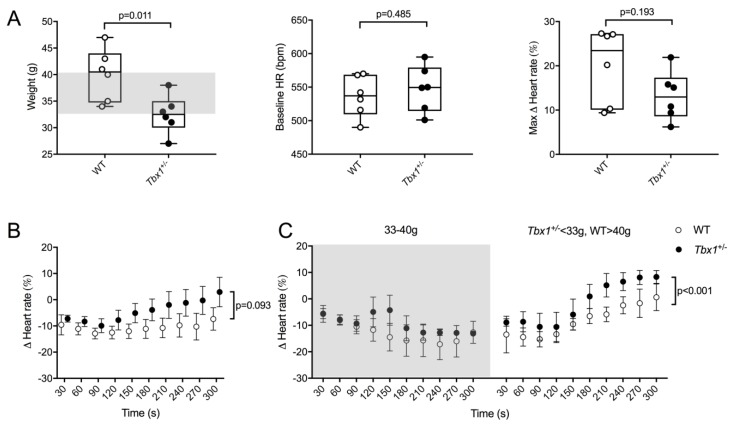
(**A**) Box and whiskers plots of body weight, baseline HR and maximum HR change; (**B**) Heart rate recovery profile following administration of carbachol in 6 WT and 6 *Tbx1*^+/−^ animals (CBC 0.1 mg kg^−1^ i.p.); (**C**) Heart rate recovery profile of animals categorised by weight. Shaded area represents range in between median weights of WT and *Tbx1*^+/−^ animals.

Diminished vagal tone can predispose to ventricular arrhythmias [29,30] as can altered balance of sympathetic and parasympathetic inputs [31]. We therefore assessed whether vagal modulation of HR was affected in *Tbx1* adult mutants. Because *Tbx1^neo2/−^* embryos die at birth from cardiovascular defects, the only available option relevant to 22q11.2DS was to perform functional analysis on adult *Tbx1^+/−^* animals. *Tbx1^+/−^* mice were significantly smaller than their WT littermates (32.5 ± 1.5 g vs. 40.0 ± 2.0 g; *p* = 0.011, Figure 4A). There was no difference in baseline HR or maximal reduction in HR over 5 min after administration of CBC (0.1 mg kg^−1^ i.p.) (Figure 4A).

Heart rate recovery was assessed by calculating the percentage difference in heart rate relative to baseline at 30 s intervals after CBC administration (Figure 4B). Heart rate recovery appeared impaired in *Tbx1^+/−^* animals, but this was not statistically significant on mixed level modelling with genotype alone as a fixed-effect variable (*p* = 0.093). There is a difference in heart rate recovery profile in *Tbx1^+/−^* and WT animals depending on their weights (Figure 4C). The heart rates of smaller *Tbx1^+/−^* and larger WT animals recovered much quicker compared to animals of average weight. This result could be due to the partially penetrant phenotype with greater reduction in vagal innervation in smaller *Tbx1^+/−^* animals. A faster heart rate recovery may reflect an increase in relative sympathetic preponderance in sympathovagal tone. Based on our preliminary work additional studies are warranted on larger sample sizes and with additional stresses and measurement modalities.

## 4. Discussion

In this study, we have described a new, dose-dependent, function for the T-box transcription factor Tbx1 in controlling vagal innervation of the heart. We found that Tbx1 regulates epibranchial ganglion positioning, cNCC migratory paths and subsequent vagal nerve projections to the heart. We also found that Tbx1 controls cardiac innervation in a non-cell autonomous fashion and that *Tbx1^+/−^* mutants show evidence of subtle abnormal vagal and muscarinic regulation of heart rate compared to their littermate controls.

The development of epibranchial placodes, and more generally all cranial sensory ganglia, is highly dependent on how NCCs develop and migrate [12]. Conversely NCCs which enter the heart to form parasympathetic cardiac ganglia follow tracts laid down by the vagus nerve [32]. It has also been demonstrated that afferent nerve pathways, i.e., cranial sensory ganglia derived from epibranchial placodes, provide a scaffold to coordinate the directional development of its efferent counterpart [33,34]. In our study, we have shown that all three components in this process are affected. Firstly, cNCCs have not migrated properly in *Tbx1^neo2/−^*. Secondly, vagal innervation to the heart is deficient in *Tbx1^+/−^* and *Tbx1^neo2/−^*. Thirdly, cranial sensory ganglia X and IX are fused in *Tbx1^neo2/−^*. We also observed that axon projections are detectable in E15 *Tbx1^neo2/−^* while no or poor NCC migrated into their outflow tract at earlier stages (E12.5). One possibility is that the remaining nerve fibres could be of sympathetic origin. Although cardiac innervation is mostly parasympathetic at E15.0, some sympathetic axons can be detected [35]. Another option is that some parasympathetic fibres have still colonised the heart, leaving open the possibility that residual NCCs make that contribution.

One of the most remarkable properties of *Tbx1* loss of function is that different embryonic structures have different sensitivity to *Tbx1* dosage [15]. In that sense, the use of a combination of hypomorphic alleles of *Tbx1* helped us appreciate that cranial sensory ganglion patterning is more sensitive to *Tbx1* dosage than cNCC migration into the outflow tract. Indeed, while cNCC migratory paths to the heart seem unaffected in *Tbx1^+/−^* animals (Figure 2), cranial nerves IX and X were abnormally patterned in *Tbx1* heterozygotes [10] Previously, using a *Sox10* in situ hybridization probe, we labelled the cranial nerves in *Tbx1^+/−^* embryos. Whereas two discrete streams of cells were observed in wildtype embryos, these streams were partially or totally fused in *Tbx1^+/−^* embryos [8]. Likewise, Karpinski et al. quantified this phenotype and found that fusion of cranial nerves IX/X occurred in 62% of *Tbx1^+/−^* embryos [10]. In addition, cNCC migration in *Tbx1^+/−^* embryos does not seem to be altered (Figure 2B). These results seem to show that vagal nerve deficit in *Tbx1^+/−^* animals depends mostly on cranial sensory ganglion patterning and to a lesser extent upon cNCC migration into the outflow tract. In agreement with this finding, we found that *Tbx1^+/−^* animals presented with a modest impairment of vagal function compared to their wildtype littermates, supporting the idea that in the adult fully developed cardiac vagal innervation depends critically on cranial nerve patterning. 

In relation to our findings, the 22q11DS adult clinic in Toronto has reported an increased incidence of sudden deaths in adults with the syndrome, with post-mortem reports highlighting cardiac sudden death as the most likely cause. This was irrespective of patients having no congenital heart defect, an insignificant heart defect (i.e., not requiring surgery), or required surgery for heart defect [36]. In this study, two (7.7%) of the 26 patients without major congenital heart disease or schizophrenia experienced unexplained death, and in the over 18 years old population sudden death is the most common cause of mortality in this condition [37]. The presence of tetralogy of Fallot may increase the risk [38]. Furthermore, it is known that 22q11.2DS patients have a normal sympathetic drive, even if they are hemizygous for the catecholamine degradation enzyme COMT [39]. Based on the results in mice, any such clinical effects are likely to be minor, and only of importance in the context of other stresses associated with the syndrome or concomitant illness. We note that, in mice, loss of the *Nhlh1* gene leads to a reduced parasympathetic drive and an associated predisposition to arrhythmia [29]. In humans, altered parasympathetic regulation of rhythm has also been considered an arrhythmogenic influence [40,41,42].

The pharyngeal surface ectoderm (PSE) was described as a key signalling centre from where TBX1-driven pathways regulate cNCC migration and pharyngeal arch artery (PAA) development. Conditional deletion of *Tbx1* in the PSE was able to recapitulate the PAA defects found in almost all of *Tbx1^+/−^* embryos, as well as the NCC migration defects associated with arch artery and cranial nerves phenotypes [43]. Indeed, cranial nerves IX and X were merged or fused in 100% of *Tbx1* PSE-conditional embryos [8]. It is therefore likely that *Tbx1* expression in the PSE is crucial not only for PAA development but also for placodal and ganglion positioning. The Pharyngeal apparatus is affected in *Tbx1^neo2/−^* embryos but in contrast to *Tbx1^−/−^* embryos which lack pharyngeal segmentation, the sixth pharyngeal arteries are sometimes present in *Tbx1^neo2/−^* embryos, as well as rudimentary fourth pouches and intact third pharyngeal arches and pouches [17]. We believe that abnormal signalling from adjacent tissue rather than pharyngeal structural abnormalities is responsible for aberrant NCC migration and placodal abnormalities. The rationale for this contention is based on our *Tbx1^+/−^* embryo study which showed atypical innervation of the heart in the context of an intact pharyngeal apparatus. Molecular perturbations affecting NCC navigation, such as disruption of neuropilin/semaphorin signalling, results in misplaced ganglia and aberrant projections [44]. Interestingly, *Sema3C* shows reduced expression in *Tbx1* mutants [45,46] and it was reported that diminished expression of *Sema3C* in *Tbx1* hypomorphs disrupted cNCC migration pattern [47]. Therefore, Sema3C could potentially participate in the cardiac innervation phenotype. In addition, mice lacking *Sema3C* die of congenital heart defects including PAA and outflow tract defects [18,48]. Furthermore, *Sema3C* mutant embryos present with a cardiac innervation phenotype similar to the one observed in *Tbx1* mutant embryos (A.C. and P.J.S., unpublished work). Because cNCCs also contribute to the development of the outflow tract, it was suggested that, unlike sympathetic precursors where semaphorins serve as repulsive cues, cardiac ganglion-destined NCCs rely on semaphorins for attractive cues during their migration. We have recently shown that cNCCs express SEMA3C and that *Sema3C* ablation in the cNCC lineage in the mouse does not alter their migration to the outflow tract [18]. Therefore, the innervation phenotype observed in *Sema3C* mutant embryos is not a cNCC migration phenotype and further studies are required to examine cranial nerve patterning in these mutant embryos.
